# Long-Lasting Effect of Perinatal Exposure to L-tryptophan on Circadian Clock of Primary Cell Lines Established from Male Offspring Born from Mothers Fed on Dietary Protein Restriction

**DOI:** 10.1371/journal.pone.0056231

**Published:** 2013-02-27

**Authors:** Elizabeth Nascimento, Omar Guzman-Quevedo, Nellie Delacourt, Raquel da Silva Aragão, Georgina Perez-Garcia, Sandra Lopes de Souza, Raul Manhães-de-Castro, Francisco Bolaños-Jiménez, Bertrand Kaeffer

**Affiliations:** 1 Departamento de Nutrição, Centro de Ciências da Saude, Universidade Federal de Pernambuco, Recife, Pernambuco, Brazil; 2 Unité Mixte de Recherche-1280, Physiologie des Adaptations Nutritionnelles, Institut National Recherche Agronomique, Université de Nantes, France; 3 Departamento de Anatomia, Centro de Ciências Biologicas, Universidade Federal de Pernambuco, Recife, Pernambuco, Brazil; State University of Rio de Janeiro, Biomedical Center, Institute of Biology, Brazil

## Abstract

**Background & Aims:**

Maternal undernutrition programs metabolic adaptations which are ultimately detrimental to adult. L-tryptophan supplementation was given to manipulate the long-term sequelae of early-life programming by undernutrition and explore whether cultured cells retain circadian clock dysregulation.

**Methods:**

Male rat pups from mothers fed on low protein (8%, LP) or control (18%, CP) diet were given, one hour before light off, an oral bolus of L-tryptophan (125 mg/kg) between Day-12 and Day-21 of age. Body weight, food intake, blood glucose along with the capacity of colonization of primary cells from biopsies were measured during the young (45–55 days) and adult (110–130 days) phases. Circadian clock oscillations were re-induced by a serum shock over 30 hours on near-confluent cell monolayers to follow PERIOD1 and CLOCK proteins by Fluorescent Linked ImmunoSorbent Assay (FLISA) and period1 and bmal1 mRNA by RT-PCR. Cell survival in amino acid-free conditions were used to measure circadian expression of MAP-LC3B, MAP-LC3B-FP and Survivin.

**Results:**

Tryptophan supplementation did not alter body weight gain nor feeding pattern. By three-way ANOVA of blood glucose, sampling time was found significant during all phases. A significant interaction between daily bolus (Tryptophan, saline) and diets (LP, CP) were found during young (p = 0.0291) and adult (p = 0.0285) phases. In adult phase, the capacity of colonization at seeding of primary cells was twice lower for LP rats. By three-way ANOVA of PERIOD1 perinuclear/nuclear immunoreactivity during young phase, we found a significant effect of diets (p = 0.049), daily bolus (p<0.0001) and synchronizer hours (p = 0.0002). All factors were significantly interacting (p = 0.0148). MAP-LC3B, MAP-LC3B-FP and Survivin were altered according to diets in young phase.

**Conclusions:**

Sequelae of early-life undernutrition and the effects of L-tryptophan supplementation can be monitored non-invasively by circadian sampling of blood D-glucose and on the expression of PERIOD1 protein in established primary cell lines.

## Introduction

Early-life stressors such as maternal undernutrition, overnutrition, hypercholesterolemia, corticosteroid therapy, uteroplacental insufficiency, or hypoxia program metabolic adaptations that initially favor survival but are ultimately detrimental to adult health. In laboratory rodents, low-protein diet during gestation and lactation has been known to reduce the life expectancy of offspring [1]. The maternal protein restriction (5–8% as compared to 18–20% in normal diet) in the rat model of In Utero Protein Restriction is one of the most extensively explored model. The low-protein fed mothers give birth to growth-restricted offspring [2], [3], and when suckled by their mothers maintained on the same low-protein diet, they remain permanently growth-restricted, despite being weaned on a normal diet [4]. Also, early-life undernutrition is associated with higher blood tryptophan levels [5], brain serotonin [6] and impairment of the serotonergic control of feeding in female adult rats [7]. Recently, we have shown that circadian clock of the hypothalamus is altered in young rats subsequently to perinatal undernutrition [8], however there is no proof that this dysregulation exists in other tissues as well. In rodents, the emergence of circadian clock outputs occur during the first 2 or 3 weeks after birth [9]. The pre and postnatal developments of the molecular clockwork in the rat liver proceed gradually with clock transcript oscillations well-organized after 30 days of life [10]. Early rhythm is entrained by the rhythm in breast feeding and care of the newborns [11]. Apparently, before weaning, peripheral clocks’ setting by the feeding regime may prevail upon entrainment by the suprachiasmatic nuclei. Some potentially entraining substrates, like melatonin which derives from L-tryptophan, may be delivered in milk [11]. From human studies, we also know that the circadian rhythm of tryptophan in breast milk affects the rhythms of 6-sulfatoxymelatonin and sleep in newborn [12], [13] and that infant formulas supplemented in L-tryptophan during the night can alter the expression of genes in cerebellum of nursing rat neonates [14]. It has been found that acute supplementation with tryptophan show transitory increase of melatonin plasma levels [15] as well as alteration in insulin secretion [16].

Several interventions (dietary or pharmacological) to reduce the long-term sequelae of early-life programming effects of several stressors have been used in animal models. The administration of folic acid with a low-protein diet during pregnancy prevents the altered phenotype and epigenotype in rat offspring [17], and administration of a diet rich in methyl donors prevents the transgenerational increase in obesity in agouti yellow mice [18]. Some works underline that the timing of such interventions can be crucial. For instance, neonatal leptin treatment which reverses the programming effects of prenatal undernutrition can be reversed with leptin treatment between Day-3 and Day-13 [19]. Here we apply L-tryptophan supplementation from Day-12 of age because Coupé et al [20] have identified extensive changes in gene expression of neurodevelopmental process related to cell differentiation and cytoskeleton organization, in the hypothalamus of rat pups born from low protein-fed mothers. As shown on adult rats [21], a daily bolus of L-tryptophan during 7 days enhances serotonin levels over a 24 hour period, and produces an advance in the peak of serotonin in both plasma and different brain regions. Long-term influence of a daily bolus can be studied on the feeding pattern, growth curves as well as on plasma D-glucose which has been described to follow a circadian rhythm during the development of obesity in rats [22]. Restricted feeding by providing a single meal at the same time each day is changing the daily profiles of PERIOD1 and PERIOD2 protein expression in brain nucleus of rats [23]. To determine whether these alterations can be measured on somatic cells accessible by non-invasive means, we have chosen to establish primary cultures of rat tail. Somatic cells like fibroblasts can be synchronized by a serum shock to re-induce clock gene expression [24] and they are believed to harbor a complete set of clock genes, retaining a function similar to the one observed in the subject [25], [26]. Moreover, primary cultured cells are easily amenable to survival under amino acid-free conditions to follow the microtubule-associated-protein light chain 3b (MAP-LC3B) which is currently the only molecular marker available for following the autophagosome in cells [27], [28], [29].

In this paper we have demonstrated a long-lasting effect of perinatal exposure to L-tryptophan on the blood D-glucose profile of male rats during the young and adult phases. On established primary cell lines, the expression of PERIOD1 protein after serum shock synchronization were different between L-tryptophan and undernourished saline groups with their controls. However, the capacity of colonization at seeding was left unchanged suggesting that developmental metabolic programming related to longevity was not reversed by our tryptophan supplementation.

## Materials and Methods

Studies on rats were realized according to the rules of the Nantes animal experimental unit (in compliance with the European Communities Directive of 24 November 1986 (86/609/EEC) and the Principles of laboratory animal care (NIH publication no. 85–23, revised 1985)). The protocol was approved by the « Comité d’éthique pour l’expérimentation animale, Pays de la Loire, France » under number 06 (March 20^th^, 2011; CEEA.2010.38). Animals were euthanized by carbon dioxide exposure.

### Diet, Animal Care and Experimental Design

Ten virgin female Wistar rats were used at the beginning of study (8 weeks, weighing 200–224 g purchased from Janvier, Rennes, France). On arrival, the rats were housed either under a photoperiod 12 h light/dark cycle (lights on at 08∶00 to 20∶00 h) or a 12 h dark/light (period from 20∶00 to 08∶00 h) reversed cycle. All handling during the dark period was done under dim red light (<2 lux), in a temperature-controlled (21±1°C) and air conditioned housing room (relative humidity: 60±10%). Animals were kept undisturbed for 2 weeks for adaptation. After confirmation of mating by visualization of spermatozoa in vaginal smears, the dams were housed individually and randomly assigned as low-protein (LP) and control (CP) groups. During all experiments, the animals were maintained with diet and water *ad libitum.* The composition of diets is shown in **Table 1**. Isocaloric (18 g% protein) or low-protein (8 g% of protein) diets were offered during gestation and lactation. Timing of delivery, litter size and pup weight were recorded at birth.

**Table 1:** Composition of low protein (8 g% protein) and control (18 g% protein) diets.

	Diets (g/kg)			Diets (g%)	
Ingredient	Low protein	Control	Nutrient	Low protein	Control
Casein (80%)	100	220			
Cornstarch (88%)	610	510	Protein	8.0	18.0
Sucrose	120	100	Carbohydrates	65.6	54.8
Soybean oil	70	70	Lipid	7.0	7.0
Fiber	50	50	Cellulose	5.0	5.0
Mineral Mix (AIN-93G)	35	35	Mineral Mix (AIN-93G)	3.5	3.5
Vitamin Mix (AIN-93G)	10	10	Vitamin Mix (AIN-93G)	1.0	1.0
L-methionin	2.5	3.0	L-methionin	0.2	0.3
Choline bitartrate	2.5	2.5	Choline bitartrate	0.25	0.25
Tert-butylhydroquinon (TBHQ) mg	8	14	Tert-butylhydroquinon (TBHQ) mg	0.8	1.4
Energetic value				357.4	352.6
% energy by protein				9.0	20
% energy by carbohydrate				73	62
% energy by lipid				18	18

The diets were isoenergetic and isolipidic. The quantity of reduced protein was replaced by carbohydrates.

The litters were homogenized for 5–6 males: 3–2 females. The sex was judged according to whether the ano-genital distance was less (female) or greater than (male) around 2.5 mm. The daily records of weight and food (7 h–9 h) allowed to calculate body weight gain, food intake and energy efficiency of dams, energy and protein intake during gestation and lactation. At Day-21, the offspring were weaned and received commercial diet for rodents (Standard diet AO4 16% protein; 4% fibers; 5% minerals and vitamins; 12% humidity and 60% glucides; 2.9 Kcal/g°) until the end of experiments.

Beside neurophysiological consideration [20], we had chosen Day-12 as the first day allowing pup’s oral gavage in Wistar rat; this first day may be different with other rat strains. From Day-12 to Day-21, every pup was administered one hour before lights off with a single bolus either of L-tryptophan (125 mg/kg; BioUltra, #93659, Sigma, France) or similar volume of vehicle saline solution (NaCl) by gavage needle. Experimental groups were abbreviated either low protein tryptophan (LPT) and low-protein saline (LPS) or control tryptophan (CTT); control saline (CTS);. Prior to bolus administration, pups were weighed to calculate the exact injection volume to be administered.

### Measurement of Food Intake

Pups aged 28–30 days were housed individually in metabolic cages (Charles River). Standard laboratory chow (SAFE, Augy, France), presented in powder form, was available *ad libitum* from a hopper recessed in to the front wall of the cage eliminating fouling of the food with urine and feces. Access to food was restricted to a horizontal slot in the hopper that allowed the rat to eat but not to remove the food. A hollow in the front portion of the hopper retained any food spilled out from the hopper. Water was dispensed from a bottle fixed to the front wall. After a habituation period of 7 days, during which the animals attained a stable pattern of feeding, food intake was monitored every 4 h for the next 3 consecutive days.

### Total Blood D-glucose

Rats aged 40–50 days and 110–120 days were sampled at the tail of one blood drop to determine the total blood glucose by Accu Chek® Active (Roche-Diagnostics GmbH, Mannheim, Germany).

### Biocollection of Primary Cell Lines

Rats aged between 45–55 days and 110–130 days, were aseptically sampled at the tip of the tail (1 mg tissue) at the end of the blood sampling cycle. The primary cells established between 45–55 days will be referred as « young » or “50” days and the cells established between 110–130 days will be referred as “adult” or « 120 days ». The young phase was chosen because it corresponds to the “hyperphagic” phase of undernourished rat pups described by all authors in the field [30]. The adult phase was chosen according to the average mating age of male rat (between 56 and 70 days) and well before the onset of obesity which is described to occur at 17 months [31]. The biopsy was briefly exposed to Javelle water, rinsed twice in a large volume of phosphate-buffered saline solution before exposure to Trypsin-EDTA during 15 min at 37°C. Cellular aggregates were mechanically disaggregated by vigorous pipetting (20 times). Cellular suspensions were layered on top of a cushion of 3 ml DMEM +20% fetal calf serum and centrifuged at 1,300×g for 3 min at room temperature. The cellular pellet was resuspended in 10 ml DMEM medium +10% fetal calf serum, amphotericin-B (1/1000) and gentamicin (100 µg/50 ml) and inoculated in 25 cm^2^ flask (Nunc®) in an humidified incubator (37°C, 5% CO_2_). Within three to five days, colonies of active cells were seen. In flask inoculated with fast-growing cells, confluency was reached within a week. Cells were resuspended by trypsinization and used to prepare a cryotube (in 95% fetal calf serum with 5% DMSO, stored in a Nalgen box before being kept permanently at −70°C), and to inoculate tissue culture dishes (LabTek, P-96 or 25 cm^2^ flask).

### Capacity of Colonization at Seeding, Adhesion and Phenotypes of Primary Cells

Freshly trypsinized primary cells from rat tail were inoculated on conventional tissue culture plastics (25 cm2 flask, Nunc®). A week later, primary cultures with actively growing cellular colonies were enumerated to calculate the capacity of colonization at seeding, and subcultured to establish cell lines. On Cytoo chambers (Starter kit; [32], suspension of 60,000 cells from 4 representative fibroblast cell lines were inoculated and left to attach 20 min before changing the medium and allowing overnight adhesion at 37°C, 5% CO2. Cells were fixed and immunolabeled for tryptophan-hydroxylase [33] and Hoechst 33258.

### Time-series Experimental Design

Rats were housed in two separate chambers and blood sampled every 4 h between Zeitgeber time (ZT) 0 to 12 and 12 to 24 h. The values obtained on both groups at ZT-12 were not statistically different. Established primary cells were cultured in various vessels (25 cm^2^ flask, Nunc, P-96 or LabTek) to obtain time-series of cellular monolayers for immunodetection or RT-PCR every 6 h over 30 hours.

### Selection of Biomarkers, Source and Specificity of Primary Antibodies

Period1 is an immediate response gene involved in the quick resetting of the circadian clock (Rabbit polyclonal antibody, Santa-Cruz sc-25362). We targeted CLOCK and PERIOD1, both involved in circadian rhythms. The transcription factor CLOCK was detected by antibodies from Santa-Cruz, sc-25361. CLOCK harbors a Histone-acetyl-transferase activity, and histone acetylation is thought to play a key role in the effects of early nutrition on gene expression, possibly mediating the long-term effects of early nutrition (nutritional imprinting). Anti-tryptophan-hydroxylase (TPH, Santa-Cruz, sc-30079) antibody was used as tryptophan-hydroxylase is the first and rate-limiting enzyme in the biosynthesis of serotonin. *In situ* detection of microtubule-associated protein light chain 3b (MAP-LC3B) by primary antibodies (Santa-Cruz (sc-28266) has been recommended when this protein constitutive of the autophagosome is overexpressed during progressive autophagy [34]. Rabbit polyclonal anti-survivin (Santa-Cruz, sc-10811) antibody was used to characterize non-apoptotic status of our cells, as survivin is a member of the inhibitors-of-apoptosis protein (IAP) family. Antibodies were used in serial dilution in confocal microscopy according to manufacturers’ requirements.

### Immunocytochemistry and Fluorescent Linked ImmunoSorbent Assay (FLISA)

Fixed cell monolayers were rehydrated by overnight incubation in phosphate buffered saline solution without Ca++ and Mg++ (PBS0). Incubations of primary antibodies were carried out overnight at 4°C in PBS0 containing 0.2% bovine serum albumin (weight/volume; fraction V, Eurotech). After 3 washing cycles with PBS0, cell preparations were incubated with Hoechst 33258 and secondary antibodies (Goat-anti-rabbit-Alexa-568, Molecular Probes) during one hour at 37°C in PBS0 containing 0.2% bovine serum albumin. After 3 washing cycles with PBS0, cellular preparations were mounted into ProLong Gold (In Vitrogen) and visualized under Leica videomicroscope (x 40 magnification) or Zeiss apotome microscope (x 63 magnification). For quantification, preparations were observed under a Leica fluorescent video microscope (x 40 magnification) with Metamorph software. The intensity of labeling by the primary – secondary antibodies complex was normalized by the total surface of the cellular body at the best plane of acquisition by densitometry with ImageJ 1.42. software as described [35].

### Microscopic Detection of Transduced LC3B-FP during Autophagosome Formation

Fluorescent Protein fused to MAP-LC3B transduced on primary cells with a baculovirus vector is also a well accepted approach to monitor autophagy whereby the appearance of fluorescent puncta are indicative of the recruitment of MAP-LC3B to the forming autophagosomes [27], [28], [29]. Autophagosome formation was detected utilizing the Premo Autophagy Sensors (LC3B-FP) BacMam 2.0 kit (Invitrogen, Carlsbad, CA, USA), according to the manufacturer’s instructions. Prem Autophagy Sensors (LC3B-FP) BacMam 2.0 kit allows transduction of cultured mammalian cells with an MAP-LC3B-fluorescent protein chimera (LC3B-FP), and a negative control fluorescent LC3B protein containing a mutation that renders the protein unable to be processed to form MAP-LC3B-II-FP (LC3B(G120A)-FP. Transduction occurs via an insect Baculovirus vector containing a mammalian promoter. Transduced cells were cultured and treated in the same way as described above. Incorporation of MAP-LC3B-II-FP in cytosolic vacuole was vizualized with a Leica fluorescent videomicroscope (x 40 magnification) or an Apotome Zeiss Microscope (x 63 magnification). Induction of vacuolization in cells was visualized by phase contrast microscopy at 100× magnification.

### Quantitative RT-PCR Experiments

We designed forward and reverse primers with Beacon Designer or PerlPrimer [36] software; the specificities were assigned independently on line with the Blast software.

Total RNA was extracted from tryptophan and saline treated fibroblasts collected at 0, 6, 12, 18, 24, and 30 h after a serum shock using the Trizol reagent (Invitrogen, Cergy Pontoise, France), treated with DNAse (RNAse free) for 30 min at 37 °C (Promega). The quality was checked on agarose gels and the quantity determined using a NanoVue™ Plus SpectrophotometerGE Healthcare at 260 and 280 nm. Afterwards, 1 µg of purified RNA was reversed-transcribed using the reverse transcription system (Promega) according to the manufacturer’s instructions. Real-time PCR was performed to measure relative mRNA expression in a Bio-Rad iCycler iQ system using the iQ SYBRGreen Supermix PCR kit (Bio-Rad Laboratories) and specific primers. PCR reactions (15 µL) were assayed in triplicate on a 96-well heat-sealed PCR plate (Thermo Scientific). Each reaction contained 7,5 µL SYBR green Supermix, 1,5 µL of forward and reverse primers, and 5 µL of cDNA (1∶40 dilution). PCR parameters were: an initial denaturation step of 5 min at 95 °C followed by 45 cycles of 30 s at 95 °C and 30 s at 60 °C. The primers used for the amplification [8] are: *Bmal1* forward 5′CAATGCGATGTCCCGGAAGTTAGA3′, reverse 5′TCCCTCGGTCACATCCCTGAGAAT3′; *Period1* forward 5′TTCGGAGCAGGCAGGTGTC3′, reverse 5′GGCAGGCGAGATGGTGTAGTAG3′ and *18S* forward 5′GATGCGGCGGCGTTATTC3′, reverse 5′CTCCTGGTGGTGCCCTTCC3′ as housekeeping gene. Relative expression levels of *Bmal1* and *Period1 mRNAs* was calculated using the comparative Δ*C*_T_ method [37]. Absolute cycle number at threshold for 18S was unchanged by tryptophan treatment.

### Data and Statistical Analyses

On data obtained from immunofluorescence imaging, normality of distribution of the intensity of a specific labeling was tested on 30 cells at a 5% level according to Kolmogorov test. Concerning, FLISA values, experimental results are expressed as means +/− s.e.m. With n = four-six cell monolayers per time point. Each cell monolayer was from a different rat.

To test for the presence of circadian rhythms, time series data were first analyzed by three-ways ANOVA using the on line available R package [38] based on classic statistics [39]. All tests were two-tailed and the significance level was set at 5% level. There is no consensus to analyze rhythms, however we have used the methodology of Exploratory Data Analysis (http://www.itl.nist.gov/div898/handbook/index.htm) and TSA Cosinor software (Expertsoft technologies, Esvres, France).

## Results

### Effects of Protein Restriction during Gestation and Lactation on Body Weight, Food Intake and Energy of Dams

Body weight and body weight gain of LP and CP dams were not different both at the beginning and at the end of experimental observation (**Table 2**). Furthermore we did not observe any alteration in the duration of gestation, the sex ratio (LP = 6.0±1.4; 5.4±1.5; males and females respectively, CP = 6.6±0.5) nor on the litters weight. However, the litters size of LP group was significantly lower than the controls (**Table 2**, p = 0.021).

**Table 2:** Gestational performance of dams fed on low-protein or control diet.

Parameters	Groups		P*
	Low- protein (n = 5)	Control (n = 5)	
Body weight initial (g)	228.8±7.0	234.8±20.13	0.543
Body weight gain (g)	133.9±52.1	164.0±22.8	0.272
Body weight end (g)	362.7±48.9	398.8±34.7	0.215
Duration of gestation	21.0±0.6	20.8±0,4	0.568
Litters size	11.2±1.6*	13.6±0,9	**0.021**
Litters weight (g) at 1 day of life	57.4±3.9	52.7±7.9	0.102

* Unpaired Student’s t-test. Overall results are means ±SD, *<0.05.

The protein intake (g/day) of LP dams was approximately 44% lower than the CP (LP = 1.6±0.13; CP = 3.7±0.4). No difference between the mean body weight of rat pups at birth was found. This fact can be related to the equivalence of food or protein intakes of all mothers throughout gestation (**Table 3**). However in agreement with previous studies, alterations in food intake and body weight were observed during lactation. During lactation, the mean food intake (g) of LP dams was of 59% of intake of CP dams (LP = 79.8±9.1; CP = 136.0±15.9). This reduction in food intake induced a deficit in energy at the end of the period around 50% (LP = 1756.5±298.2, CP = 3467.5±608.3). The protein intake of LP dams was approximately 75% of the one of CP dams.

**Table 3:** Weekly evolution of body weight gain, food intake, and energy of dams fed on low-protein or control diet during gestation and lactation.

Gestation (weeks)									
Variables	Low protein Group			Control Group					
	1^a^	2^a^	3^a^	1^a^	2^a^	3^a^		P	
Weight gain (%)	12.1	10.9	22.4	13.7	13.7	26.7		<0.001	
	±3.5^a^	±6.6^a^	±11.4^b^	±3.6^a^	±2.4^a^	±1.1^b^			
Daily Food intake(g)	24.7	20.2	21.6	18.8±3.0	21.1	23.8			
	±7.6	±4.7	±6.6		±3.5	±1.7		>0.05	
Food intake relative(g/100 g)	7.9	7.5	6.2	7.5	7.3	6.6			
	±0.5^a^	±1.5^a^	±1.3^b^	±0.8^a^	±0.8^a^	±0,2^b^		= 0.001	
Daily Energy relative(kcal/100 g)	28.4	27.5	24.8	26.6±2.7^a^	26.7	24.2			
	±1.9^ a^	±4.3^ a^	±4.2^ b^		±2.9^a^	±1.9^b^		= 0.035	
Protein relative(g/100 g)	0.6±0.05^b^	0.7±0.04^b^	0.6	1.3	1.3	1.2		>0.05	
			±0.07^b^	±0.13^ a^	±0.14^a^	±0.10^ a^			
**Lactation (weeks)**									
**Variables**	**Low protein Group**			**Control Group**					
	**1^a^**	**2^a^**	**3^a^**	**1^a^**	**2^a^**	**3^a^**		**P**	
Weight variation(g/%)	−12.7	−5.2	−3.6	1.6	−1.1	−1.2		<0.001	
	±6.5^b^	±1.8^bc^	±6.8^bc^	±4.3^a^	±3.5^a^	±3.2^a^			
Food intake(g)	26.4^d^	23.3^d^	30.0^e^	37.2 ^a b^	46.1^ b c^	52.7^c^		<0.001	
	±6.7	^e^ ±4.0	±8.3	±12.4	±3.9	±3.7			
Food intake relative(g/100 g)	6.7	10.4	13.5	10. 8	14.9	17.2			
	±1.8^d^	±2.3^e^	±2.2^f^	±1.4^a^	±0.9^b^	±1.4^c^		<0.001	
Energy relative(kcal/100 g)	23.9	37.1	48.3	39.0	53.7	63.5			
	±7.2^d^	±8.6^e^	±18.1^f^	±4.5^a^	±3.3^b^	±4.1^c^		<0.001	
Protein relative(g/100 g)	0.5^d^	0.8^e^	1.5^f^	1.9^a^	2.60^b^	3.10^c^		<0.001	
	(±0.1)	(±0.2)	(±1.0)	±0.2	(±0.2)	(±0.2)			

Two-way ANOVA followed by Bonferronís test. Data correspond to the mean ±DP of each experimental group. Different letters in same line indicate statistically significance.

The evolution of body weight, food intake and energy of LP dams **(** **Table 2** **)** showed a global deficiency in nutrients and calories, since LP dams ingest 30% less carbohydrate (LP = 52.3±6.0; CP = 74.5±8.7 g) and 40% less lipids (LP = 5.6±0.6; CP = 9.5±1.1 g) than CP dams.

The protein intake (g/100 g/day) of LP dams was approximately 44% lower than of CP dams **(** **Table 3**). The total food intake of LP dams was half of CP dams (LP = 487.9±82.8; CP = 963.2±169.0 g, P<0.01). This reduction in food intake induced a deficit in energy at the end of the period around 50% (LP = 1756.5±298.2, CP = 3467.5±608.3, P<0.001).

At the end of lactation, the dams fed on low protein has 24% less body weight than control dams (LP = 232,7±44.2; CP = 305.7±19.9). The variation of body weight recorded during the lactation period confirms this trend (LP = −22.64±4.6 g and CP = 4.45±3.88 g).

### Body Weight, Body Growth and Food Intake of Male Offspring

**Figure 1:**
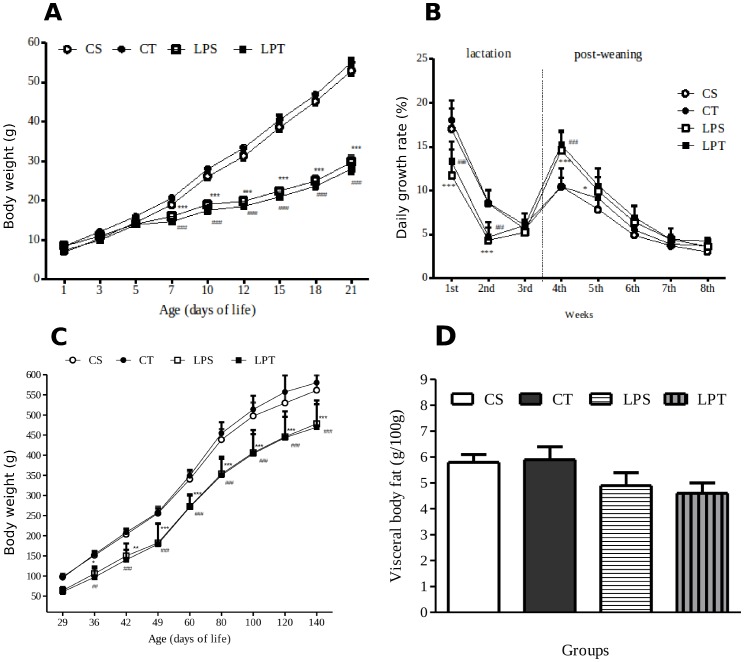
Growth of offspring and amount of visceral fat at sacrifice. Evolution of body weight (A) and daily growth rate (B) of offspring receiving a daily bolus of tryptophan or of saline solution from Day-12 to 21 of age. Evolution of body weight of offspring after weaning (C) and visceral fat at day 140 (D). Four groups of rat pups are shown referred as LPS = Low-protein saline (n = 9 ); LPT = low-protein tryptophan (n = 9). Body weight s’ gain of low protein and control groups were divergent at day-7 and remained so independently of tryptophan supplementation. Data are expressed as means and ±SEM. *P<0.05; **P<0.01; ***P<0.001 by two-way ANOVA followed by Bonferroni test. (*LPS vs CS and #LPT vs CT). The body weight of offspring (n = 42) after weaning until 140-old age remained lower until the end of experiment (C) with similar visceral fat (g/100 g) at sacrifice (D). Data are expressed as means ±SEM. CS = control saline (n = 12 ); CT = control tryptophan (n = 12 ); LPS = Low-protein saline (n = 9 ); LPT = low-protein tryptophan (n = 9). *P<0.05; **P<0.01; ***P<0.001 by RM two-way ANOVA followed by Bonferroni test (*LPS vs CS and #LPT vs CT.).

Body weight gain of low- protein and Control groups were divergent at day-7 independently of tryptophan supplementation (**Figure 1 A**). At weaning, the pups of LP dams were 47% lighter than the control ones (LPT = 27.5±1.4; LPS = 28.3±1.4; CTT = 53.9±3.4; CTS = 53.1±1.3) indicating growth retardation. The daily body weight gain was maximum at the 1^st^ post natal week and decreased thereafter (**Figure 1B**). The daily body weight gain was never higher after post weaning (4^th^ and 5^th^ week) with all groups from low-protein diet.

A daily bolus of L-tryptophan between D-12 and D-21 did not alter the catch-up growth of offspring from weaning to final growth (around ×530% with LP and ×370% with CP). Our results confirm that the post-weaning period (22–40^th^ days of life) is crucial in the compensation of body weight gain of pups suffering of growth retardation. These data are consistent with the temporary hyperphagy previously demonstrated [40]. However the body weight of low protein group (saline or tryptophan) in our experiment remained lower until the end of observations (**Figure 1C**) but the groups did not differ in visceral fat (**Figure 1D**).

**Figure 2:**
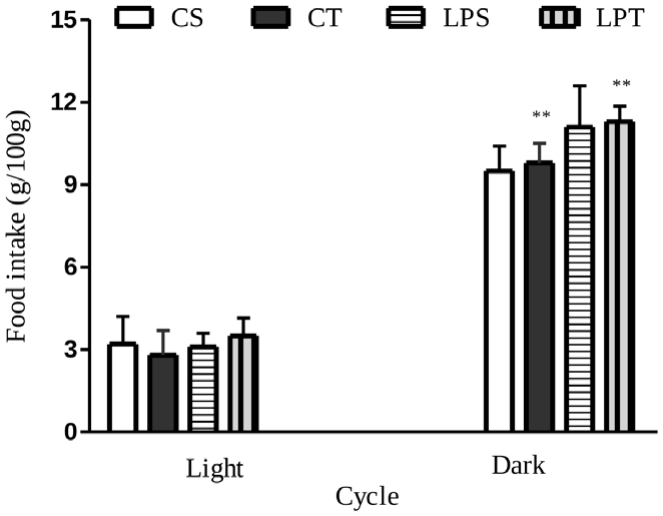
Relative food intake of pups between day-39 and day-42, from dams fed on low-protein or control diet during perinatal period. Rat pups received a daily bolus of L-tryptophan or Saline from day-12 to day-21. On rat pups from mothers fed on low protein diet, means of food intake measured in dark cycle were significantly different from control, irrespectively to L-tryptophan supplementation (Low-Protein Tryptophan (LPT, n = 9 pups); Low protein Saline (LPS, n = 9 pups); Control Tryptophan (CT, n = 12 pups), Control Saline (CS as white) *P<0.05 ***P<0.0001 by one way ANOVA followed by Bonferroni test. Data are expressed as means ±SEM (*LPS vs CS and #LPT vs CT.).

The daily food intake of rat pups measured between Day-39 and Day-42 showed that the absolute consumption of the LP group is lower than control group, irrespectively of a supplementation by L-tryptophan **(Figure S1)**. In addition, the relative food intake was higher in the LP group during dark cycle, but it was similar during light cycle (**Figure 2**).

Taken together these observations demonstrate that a daily supplementation of tryptophan between Day-12 and Day-21 did not alter up to 140 days, body weight, body weight gain, and feeding pattern of low-protein or control groups.

### Total Blood Glucose Profiles

**Figure 3:**
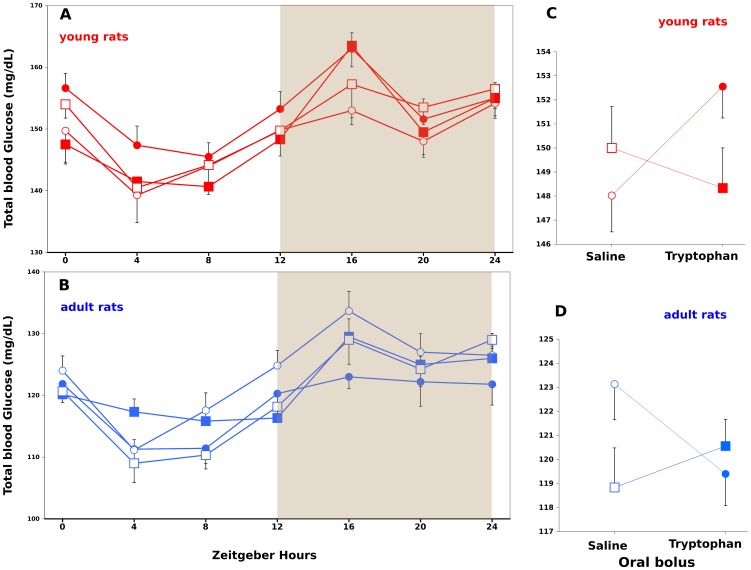
Evolution of total blood glucose over 24 hours of rats sampled during young (red) and adult (blue) phases. Rats were from mothers fed on Low protein diet (square) with bolus of L-tryptophan (filled, LPT) or without (white, LPS) and Control diet (circle) with bolus of L-tryptophan (filled, CT) or without (white, CS). Data are expressed as means ±SEM. By three-way ANOVA we found a significant effect of the Zeitgeber (Hours) on both time series (p<0.001; A & B) and a significant interaction between Diet (Low protein, Control) and daily bolus (L-tryptophan or saline) for young phase (p = 0.0291; C) and for the adult phase (p = 0.0285; D). Note that interactions between factors shown on C and D are reversed. By applying Cosinor analysis, we found that the maximum at 16 h was representative of a rhythm for the group of rats fed as control and receiving daily bolus of L-tryptophan (Fourier analysis, autospectral plot and spectral density analysis gave a maximum at 16.7 h for CT series on A).

The total blood glucose profiles were strictly different between the young and adult phases (Note that the range of D-glucose values are between 130 to 170 mg/dL for young (**Figure 3A**) and 105–140 mg/dL for adult (**Figure 3B**) phases). A significant effect of the sampling time was found for the groups of young and adult rats, p = 0.0007 and p<0.0001, respectively by 3-way ANOVA. The absence of interaction between sampling time and other factors indicates that all groups had a representative profile with the maxima of all series at 16 h. By applying Cosinor analysis, we found that the maximum at 16 h was representative of a rhythm for the group of rats fed as control and receiving daily bolus of L-tryptophan (Fourier analysis, autospectral plot and spectral density analysis gave a maximum at 16.7 h). A striking observation was the reversion between CT and CS profiles according to the phases. The CT profile (filled circle in A and B) showed the highest values in the young rats and only low values in the adult rats. The CS profile (white circle in A and B) showed low values in the young rats and the highest values in the adult rats (except at 4 hours). The profiles of undernourished rats remained between the CS and CT profiles.

**Figure 4:**
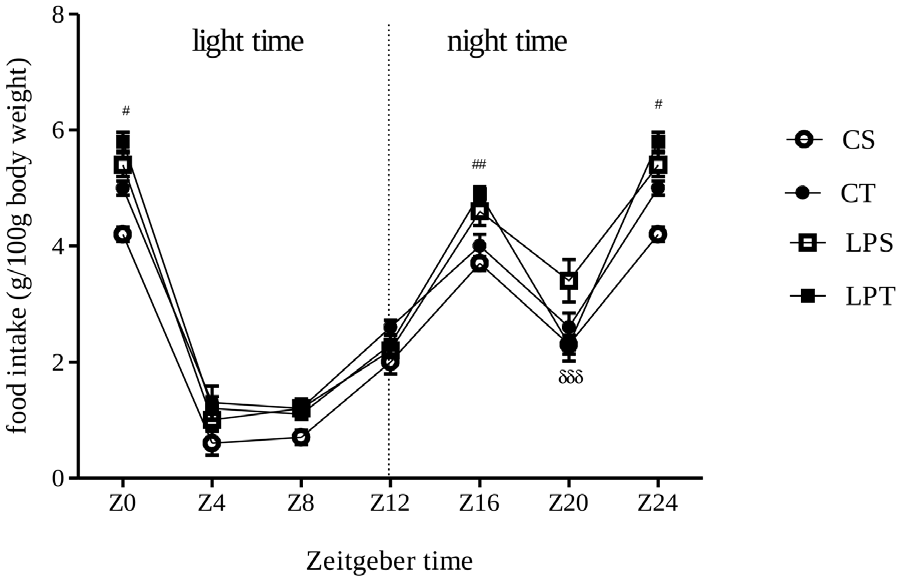
Daily percentages of food intake of rats during 4 days of observation of consumption every 4 h. The litter mate (n = 38) derived from dams fed control or low protein diet during perinatal period. The pups received L-tryptophan or saline (125 mg/kg body weight) between Day-12 and 21. Only litter mates LPT (45–55 old age) showed difference on the cycle of food intake during 4 consecutive days of observations. Data (g/100 g body weight) are expressed as means ±SEM. *^#^p<0.05 by two-way ANOVA followed by Bonferroni test.; *LPS X CT and CS; ^#^LPT X LPS.

A significant interaction between daily bolus (L-tryptophan or saline) and diets (Low protein versus Control) were found for the young rats (p = 0.0291 by 3-way ANOVA; **Figure 3C**) and for the adult rats (p = 0.0285; **Figure 3D**). These data show that the metabolic status of low protein as well as of control rats was profoundly altered by the daily bolus of L-tryptophan. By comparing **figures 3C and D**, interactions between factors were reversed indicating that we had selected 2 strictly different phases. By following food intake during 4 consecutive days of observations, we found that only LPT rats had a significantly different profile from others (**Figure 4**).

Observations from **figures 3A** **and** **4** demonstrate that the daily bolus of tryptophan had an effect on the phenotype of rat pups from the low-protein fed mothers during the young phase.

In order to explore the interaction between perinatal L-tryptophan supplementation and perinatal undernutrition of rat pups, we chose to select primary cells from the tip of the tail of every rat. The general aim was to design functional assays with living cells which may be sampled in rats and in humans.

### Capacity of Primary Cells to Adhere and Colonize Plastics at Seeding and Diversity of Phenotypes Selected from the Biopsies

**Figure 5:**
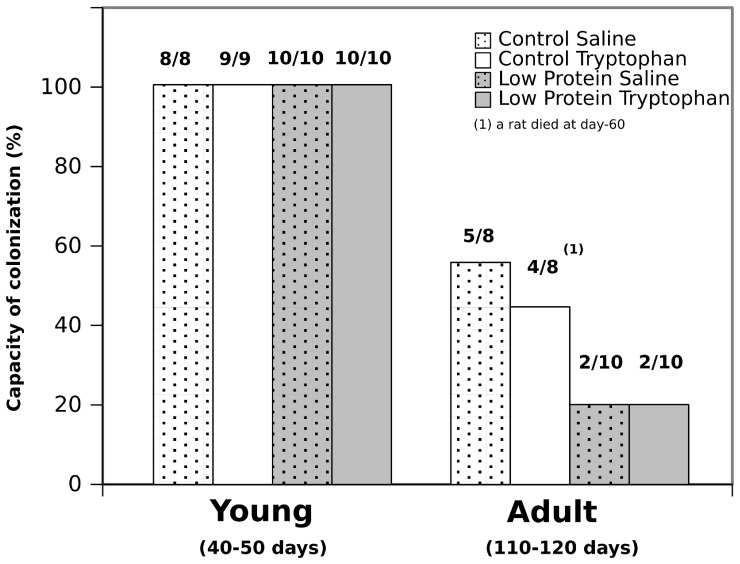
Adhesion and colonization of primary cells on conventional plastics according to age. Cells were isolated by trypsinization from tail biopsies within 7 days of culture. Identical capacity to rise primary cultures were found for rats whatever their mother’s diet and perinatal treatment during the young phase. A significant loss in the capacity of colonization (p<0.05) was found for cellular preparations obtained from undernourished adult rats whatever the perinatal treatment (with or without a daily bolus of L-tryptophan).

During the young phase, the capacity of colonization of primary cells at seeding was of 100%. All cellular preparations successfully prepared gave colonies of actively growing cells colonizing plastics within one week (**Figure 5**). However, we have found that the speed of adhesion was slower with cell preparations of the LP rats. To test the homogeneity of cellular preparation and the capacity to adhere to a substratum, we have seeded established cell lines of LP and CP groups onto Cytoo chamber starter kit. The Cytoo kit proposes different preset forms onto plastic substratum pre-coated with fibronectin. After adhesion according to manufacturer’s requirements, cells were fixed, and nuclei were revealed by Hoechst staining along with an immunostaining for the expression of tryptophan-hydroxylase. The diversity of cell phenotypes was identical between established cell lines as well as the expression of tryptophan-hydroxylase (**Figure S2**). However the number of cells able to adhere to fibronectin substratum from the low protein groups (receiving a daily bolus of L-tryptophan or of saline solution) was lower (percentage of sites with a cell: 23.6% ±7.6% than for the cells isolated from rats of control group (percentage of sites with a cell: 65.9% ±4.9%) confirming observation at the onset of primary cultures. Primary cells collected from adult rats were significantly less able to colonize plastics after one week in culture than corresponding cells from young rats (**Figure 5**).

Together these data indicate that rats derived from LP dams were less prone to give rise to primary cell cultures after the young phase, irrespectively of a daily bolus of L-tryptophan. In the following experiments, we have chosen to focus on the fastest growing cell lines derived from each group of rats. Cell monolayers were studied either after synchronization to follow the circadian expression of tryptophan-hydroxylase, PERIOD1, CLOCK or under conditions of starvation to follow the circadian expression of autophagic (LC3-B, Survivin) biomarkers and of PERIOD1 proteins.

### Expressions of Tryptophan-hydroxylase and CLOCK Proteins Over 30 Hours after a Serum Shock

We did not find any difference over time in the expression of tryptophan-hydroxylase as well as of CLOCK proteins by primary cultured cells of all experimental groups (unshown results).

### Expression of PERIOD1 Protein Over 30 Hours after a Serum Shock

**Figure 6:**
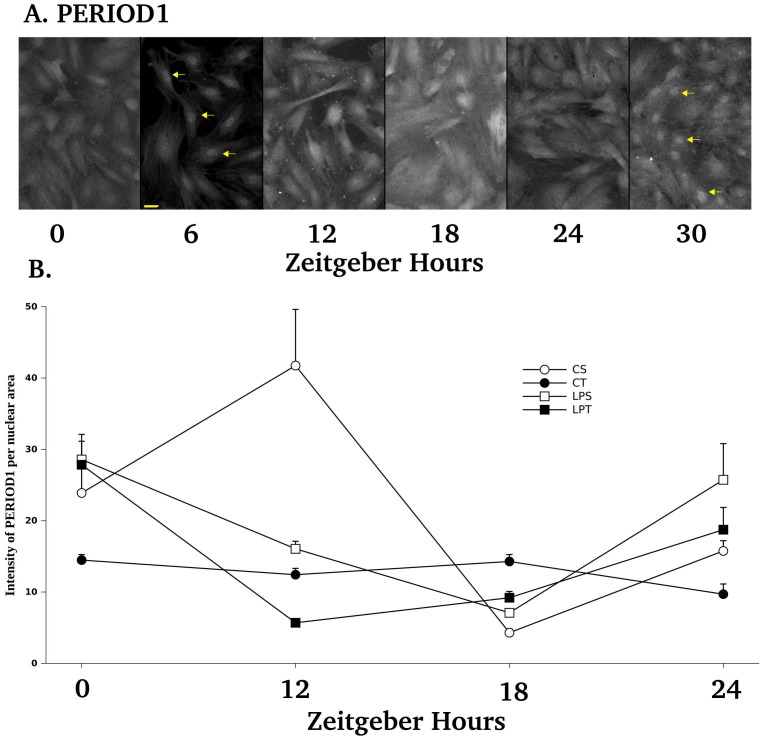
Immunodection of PERIOD1 on primary cell monolayers from young rats over 30 hours after a 2 h serum shock according to diets and tryptophan supplementation. A. Expression of PERIOD1 was found localized in the nucleus (yellow arrows) at 6 and 30 h after serum shock. Consistent observations of the nuclear localization of PERIOD1 at 6 h were indicative of correct cell synchronization by the serum shock. Re-occurence of nuclear staining at 30 h showed PERIOD1 cycling. The yellow bar at bottom of 6H plane stands for 10 µm. B. Quantification of nuclear PERIOD1 staining on confocal images of cellular monolayers by using the Hoechst staining to delineate nuclei area and to integrate PERIOD1 staining. By three-way ANOVA, we found a significant effect of diets (p = 0.0490), of daily bolus of L-tryptophan (p<0.0001) and of Hours (p = 0.0002). All factors were significantly interacting (p = 0.0148). Data are expressed as means ±SEM. A range of 31 to 74 nuclei were measured for the intensity of PERIOD1 from at least 3 cell lines per group.

Synchronization by fetal calf serum could be checked by the localization of PERIOD1 proteins in nuclear or perinuclear sites for all examined cells at 6 h (**Figure 6** **A**). Cycling expression of PERIOD1 proteins were shown by the clear-cut nuclear labeling obtained at 6 and 30 h (**Figure 6** **A**). By confocal image analysis made on the best plane of nuclear PERIOD1 labeling, we have shown that the profiles of PERIOD1 nuclear localization were different between all groups. A range of 31 to 74 nuclei were measured for the intensity of PERIOD1 from at least 3 cell lines per group (**Figure 6** **B**). By 3 Way ANOVA, we found a significant effect of diets (p = 0.049), of daily of bolus of L-tryptophan (p<0.0001) and of Zeitgeber hours (p = 0.0002). All factors were significantly interacting (p = 0.0148) indicating that PERIOD1 profiles of LPT, LPS and CT were strictly different from CS profile. In parallel we have performed by FLISA the analysis of the total intensity of PERIOD1 labeling and we did not obtain any difference in the profiles of PERIOD1 immunolabeling when integrating cytoplasmic and nuclear labeling (**Figure S3**).

### Evolution of Period1 Transcript Over 30 Hours after a Serum Shock

As shown **Figure S4**, the profiles of period1 mRNA as well as of bmal1 mRNA of primary cells collected from control-fed rats were not different from the one obtained with control-fed rats supplemented with L-tryptophan.

### Expression of Autophagic Biomarkers (MAP-LC3B and Survivin) Over 30 Hours of Starvation

**Figure 7:**
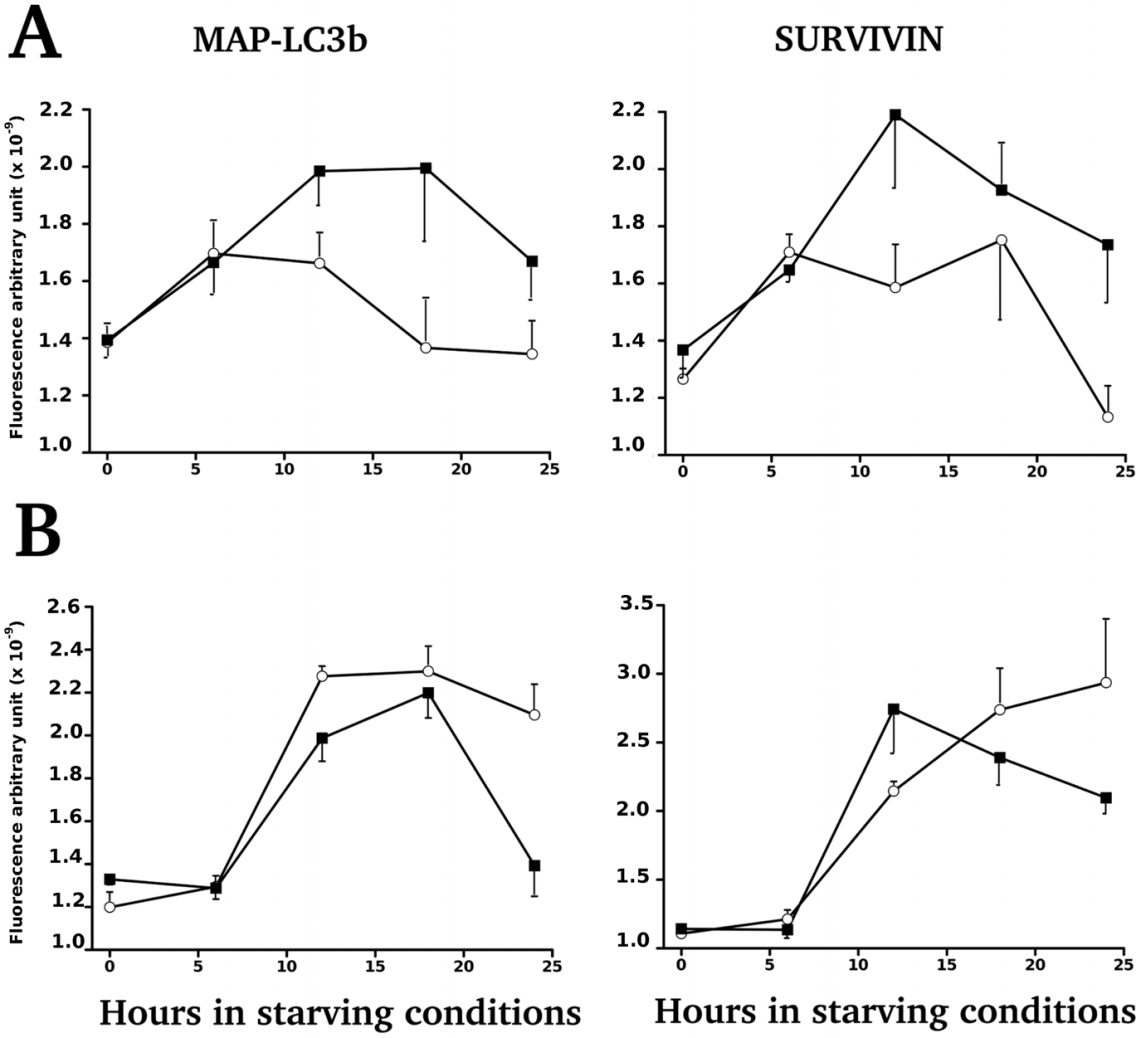
Expression of MAP-LC3B and Survivin by primary cells submitted to starvation. A. Primary cell cultures established during the young phase from rats fed either a Low protein (black square) or control diet (white circle). By two-way ANOVA, we found a significant effect of diet (p = 0.0005) and of duration of starvation (p = 0.0343) for MAP LC3B, even if Survivin expressions were following a similar trend they were not statistically significant. B. Primary cell cultures established from adult rats, fed either a Low protein (black square) or control diet (white circle). Data are expressed as means ±SEM. By two-way ANOVA, we found a significant effect of diet (p = 0.0173) and of duration of starvation (p<0.0001) for MAP LC3B with a significant interaction of both factors (p = 0.038). Only the interaction of both factors for Survivin was significant (p<0.0001).

Near-confluent cell monolayers were rinsed and exposed to phosphate-buffered saline solution supplemented with 1% fetal cal serum following Chiou et al (2011) [41]. Time series were realized to follow the level of expression of MAP-LC3B and Survivin over 2, 6, 12, 18, 24 and 30 h by using FLISA (**Figure S5**). Expression of MAP-LC3B and Survivin did not differ according to the daily bolus of L-tryptophan. Significant differences were found between cell lines isolated from rats with LP dams and from rats with control dams on the density of cells (**Figure S5**) and on the total intensity of immunolabeling (**Figure 7** **A**) during the young phase. The expression of Survivin was following a similar pattern during the young phase (**Figure 7** **A**). However the profiles of MAP-LC3B and Survivin obtained on primary cells established during the adult phase were different (**Figure 7** **B**).

In parallel experiments, we have used a baculovirus vector to study the expression of MAP-LC3B-FP in the autophagosome corresponding to the form II of MAP-LC3B. Primary cells were found to be easily infected by baculovirus construction in the range of 80–90% positive cells per culture. Representative cell lines of each group were submitted to starvation during 4 hours before recording the typical localization of LC3B in autophagosomes (**Figure S6**) or submitted to one hour serum shock followed by 4 hours starvation (unshown results). We have found that the expression of red fluorescent punta were qualitatively higher in cell lines isolated from control-fed rats than in undernourished rats (**Figure S6 C and D**) according to our quantification realized by FLISA (**Figure 7**). However, we cannot confirm the later points as the fluorescence of our baculovirus construction was quenched after 4 hours in culture by repetitive recording of fluorescence. Under starving conditions, we did not show any difference in the level of PERIOD1 immunoreactivity between these cell lines.

## Discussion

L-tryptophan supplementation was given to manipulate the long-term sequelae of early-life programming by undernutrition. We found that the effects of this dietary intervention can be monitored non invasively by circadian sampling of blood D-glucose. Expressions of PERIOD1 protein by synchronized primary cell lines established from young rats were altered according to diet and L-tryptophan supplementation. However the capacity of colonization at seeding and the adhesion potentials of primary cells were clearly altered in rats born from mothers fed on dietary protein restriction, irrespectively of the supplementation with L-tryptophan.

The body weight means of low-protein groups (tryptophan or saline) in our experiment remain lower than control groups until the end of the observation period (**Figure 1C**), but, the weight of visceral fat of undernourished offspring was similar to control group at the end of experiment (**Figure 1D**). L-tryptophan supplementation between D-12 and D-21 did not alter the catch-up growth nor the absolute food consumption of male offspring from weaning to the end of growth period (22–50^th^ days of life). The daily food intake of rat pups measured between Day 39 and Day-42 showed that the food consumption of the LP group is lower than the CP group irrespectively of a supplementation by L-tryptophan **(Figure S1)**. These observations on nursing rats with offspring are similar to results obtained in lactating sows [16]. In addition, the relative food intake was higher for the LP group during the night cycle but the relative intakes of both groups were similar during the light cycle (**Figure 2**). These data are consistent with the temporary hyperphagy that we previously demonstrated in undernourished rat pups [40].

A daily bolus of L-tryptophan between D-12 and D-21 did alter the level of D-glucose both in LPT and CPT groups **(** **Figure 3** **)**. Our D-glucose profiles displayed a maximum around 16 h Zeitgeber time like the profiles obtained on plasma D-glucose titration [22]. However a significant circadian rhythm of D-glucose oscillation was only obtained with the control-fed group receiving a perinatal bolus of L-tryptophan. Results shown on **Figures 3** **and** **5** suggest that there are a long-term effect of tryptophan supplementation on rats enduring perinatal undernutrition as well as on control-fed rats. As shown on **Figure 4**, we found a significant difference on the cycle of food intake during 4 consecutive days of observations on rats exposed to perinatal undernutrition and receiving a daily bolus of L-tryptophan. These results are suggesting that our supplementation triggered discrete phenotypic alterations. We think that our results indicate that milk formulas designed to improve sleep-wake cycles of babies have to be tested on rat models under several conditions of feeding to check for global phenotypic consequences. Beside oral gavage, L-tryptophan supplementation has to be tested from birth in formulated milk by using gastrostomized rat pups [42] or with subcutaneous or intraperitonal injections. In any case, subsequently to our experiment, testing formula fortified with L-tryptophan on cerebellum gene expression of nursing rat neonates [14] is clearly insufficient.

To explore whether our supplementation with L-tryptophan interacted with the influence of perinatal undernutrition on male somatic cells, we have selected primary cells from the tip of the tail of every rat. We have tested the capacity of these primary cells to adhere and colonize plastics (**Figure 5** **)** and the diversity of phenotypes selected from the biopsies **(Figure S2)**. Together these data indicate that adult rats derived from LP dams were less prone to give rise to primary cell cultures, irrespectively of a daily bolus of L-tryptophan. This influence of maternal undernutrition on rat pups is in line with previous works on mice reporting that SIRT1 expression was reduced and many insulin-related signaling molecules were altered [43] explaining a reduction in longevity. Tryptophan supplementation has clearly the potential to alter clock-related dysregulation but it is not sufficient to revert the reduction in longevity related to perinatal undernutrition.

A daily bolus of L-tryptophan had a profound effect on the profiles of PERIOD1 protein expression for both diets **(** **Figure 6A and 6B** **)**. Our microscopic approach is taking advantage of confocal imaging to trace the distribution of PERIOD1 in the different cellular compartments. The re-induction of PERIOD1 protein expression in our primary cells observed between 6 and 18 h was similar to the PERIOD1 reactivity described in rat brain region between 6 and 13 h [**23**]. By focusing on the perinuclear and nuclear localization of PERIOD1, we have been able to appreciate the level of synchronization of our cells (PERIOD1 can be detected during transit at the perinuclear and nuclear membranes locations) as well as the total nuclear intensity of expression according to previously described methods [23], [35]. A daily bolus of L-tryptophan had a profound effect on the profiles of PERIOD1 protein expression for both diets. These results are in line with our previous work indicating that perinatal undernutrition alters the circadian expression of period1 mRNA of hypothalamus of young rats [8].

The environmental synchronizers are integrated by response elements located in the promoter region of period genes that drive the central oscillator complex. The period genes are also members of the immediate early gene family because cells like human normal fibroblasts exposed to cycloheximide, an inhibitor of transcription, retain a response toward stressful conditions characterized by a dramatic increase in PERIOD proteins [***44***]. As shown on **Figure S4**, the expression profiles of period1 and bmal1 mRNA by cells collected from control-fed rats were not different from the ones obtained with control-fed rats supplemented with L-tryptophan suggesting that these alterations may be at the protein level, further works with cycloheximide are needed to clarify this point. The promoters of period1 and 2 genes (but not of period3) contain a cAMP-responsive element (called CRE) that binds to CREB proteins. These CRE sites are integrating the cAMP response to a wide category of synchronizers (like serotonin, glutamate, calcium ions, and light) as well as the response to a second wide category of synchronizers (like growth factors, hormones, and cytokines) acting through the extracellular signal regulated kinase leading to the mitogen-activated kinase pathways, independently of the CLOCK: BMAL1 activity [45]. We have used a serum shock to re-induce clock machinery; experiments are scheduled to explore which specific pathways are dysregulated by using molecular compounds like dexamethasone, Forskolin, dibutyryryl cAMP, phorbol-12-myristate, calcimycin, epidermal growth factor, insulin, or fibroblast growth factor.

The expression of autophagic biomarkers (MAP-LC3B and Survivin) over 30 hours of starvation **(** **Figure 7**, and for monolayer microscopic observations **Figure S5)** were suggesting that a daily bolus of L-tryptophan did not alter the autophagic machinery of primary cells but that the phenotypes derived during the hyperphagic phase from rats enduring a perinatal malnutrition had deeply altered autophagic machinery. Similar cellular phenotypes obtained during the prediabetic phase (110–130 days) did not show similar deregulation indicating that the alteration of autophagic machinery was only transient. Reversibility of molecular alterations induced in living cells by early-life nutritional stress is a major drawback to the long-term monitoring of the sequelae of early-life programming effects, especially by non-invasive means. The lower lifespan of rats whose dams fed low-protein diet during perinatal period had been shown in earlier studies [46] but the underlying mechanisms remain unclear [47]. The potential factors have been investigated as oxidative injury in key tissues [48] and telomere shortening [49], [50]. During the young phase, high blood glucose levels were indeed observed at ZT-0 in control tryptophan and control saline groups but could not be related to food intake. However, during the adult phase, we have found a shift in profiles (at ZT-16 the maxima were lower for both groups). These results indicate that there are intimate interactions between the clockwork and the cellular metabolism. In the future, we could realize epigenetic profiling of each cell line to dissect the molecular cascades altered relatively to the original rat diet. Another avenue of research is to establish primary cell lines of embryos from Low-protein or control-fed mothers to check for some difference at the onset of period1 gene regulation by CLOCK:BMAL1 activity in relation to autophagy.

In conclusion, our results demonstrate that the young phase is characterized by transient behavior and metabolic variations which can be traced at the molecular level on living cells. The general aim was to design functional assays with living cells which may be sampled in long term experiments under similar conditions as ours or with humans by non-invasive means like skin fibroblasts [26], hair follicles [51], urinary cells [52] or exfoliated cells of gastric [35], or nasal [53] epitheliums. The availability of 50 primary cell lines retaining nutritional stress-related alterations in PERIOD1 expression open the way to design functional assays on living cells on the dynamic of the circadian epigenome [54] like determining if the profile of H3K9/K14 histone acetylation [55] in fibroblasts is comparable to the one found in neurons.

## Supporting Information

Figure S1 **Daily food intake of pups between day-39 and day-42, from dams fed on low-protein or control diet during perinatal period.** Rat pups received a daily bolus of L-tryptophan or Saline from day-12 to day-21. On rat pups from mothers fed on low protein diet, daily food intake was significantly different from control, irrespectively to L-tryptophan supplementation (Low-Protein Tryptophan (LPT, n = 9 pups); Low protein Saline (LPS, n = 9 pups); Control Tryptophan (CT, n = 12 pups), Control Saline (CS as white) *P<0.05 ***P<0.0001 by one way ANOVA followed by Bonferroni test. Data are expressed as means ±SEM (*LPS vs CS and #LPT vs CT.).
(TIF)

Figure S2 **Morphology of representative primary cells, all expressing tryptophan hydroxylase (red) on Cytoo well preset with a wide array of forms.** Similar diversity of phenotypes (A to E) was found on primary cells isolated from rat submitted to perinatal undernutrition or not. Supplementation with a bolus of L-tryptophan did not alter the phenotypes.
(TIF)

Figure S3 **Total intensity of PERIOD1 proteins expressed by primary cells of young (A) and adult (B) rats.** Data are expressed as means ±SEM. Note that we did not find any difference between these cell lines (n = 4 to 6 cell lines per point).
(TIF)

Figure S4 **Evolution of mRNA expression of period1 (circle) and bmal1 (triangle) after a serum shock of cell lines obtained from at least 3 rats of the control group receiving a daily bolus of L-tryptophan (closed symbol) or a saline solution (open circle).** Expression of circadian clock Per1 (A) and BMAL1 (B) transcripts in primary cultures from tryptophan (black square) and saline (white circle) rat offspring from mother fed on control diet. The transcript levels at 6 h intervals were measured by quantititative PCR and synchronized to time 0 h by fetal calf serum. Graphs represent the relative transcriptional level of genes averaged over at least 4 independent samples isolated by offspring of 45 d old derived from dams fed control diet and supplemented or not wit L-tryptophan early 12 d old at 21 d old. Each point corresponds to the means ±S.E.M. expression levels of 4–6 cells by groups (two-way ANOVA followed by Bonferroni test).
(TIF)

Figure S5 **Expression of MAP LC3B protein in representative cultures of primary cells from rats with perinatal undernutrition or control-fed.** Autophagosomes were clearly labeled after 6 under starving conditions (yellow arrows). Note that the density of cells are equivalent up to 12 h, thereafter the density of cells isolated from the rat with perinatal undernutrition is higher than the density of control. All cultures were made of surviving cells at 30 h and were not used for quantification.
(TIF)

Figure S6 **Expression of the chimeric LC3B-FP protein after infection of primary cells by a baculovirus construction and 4-hour starvation.** Cells were isolated during the hyperphagic period from undernourished rats receiving daily bolus of L-tryptophan (A) or saline solution (B) and from control-fed rats receiving a daily bolus of L-tryptophan (C) or saline solution (D). Note that the number of autophagosomes labeled in red (yellow arrowheads) are equivalent between infected cells.
(TIF)
