# Supplementary figures and images for: Long-Lasting Effect of Perinatal Exposure to L-tryptophan on Circadian Clock of Primary Cell Lines Established from Male Offspring Born from Mothers Fed on Dietary Protein Restriction
Source: PLoS One. 2013 Feb 27;8(2):e56231. doi: 10.1371/journal.pone.0056231 (PMC3584092; doi:10.1371/journal.pone.0056231)

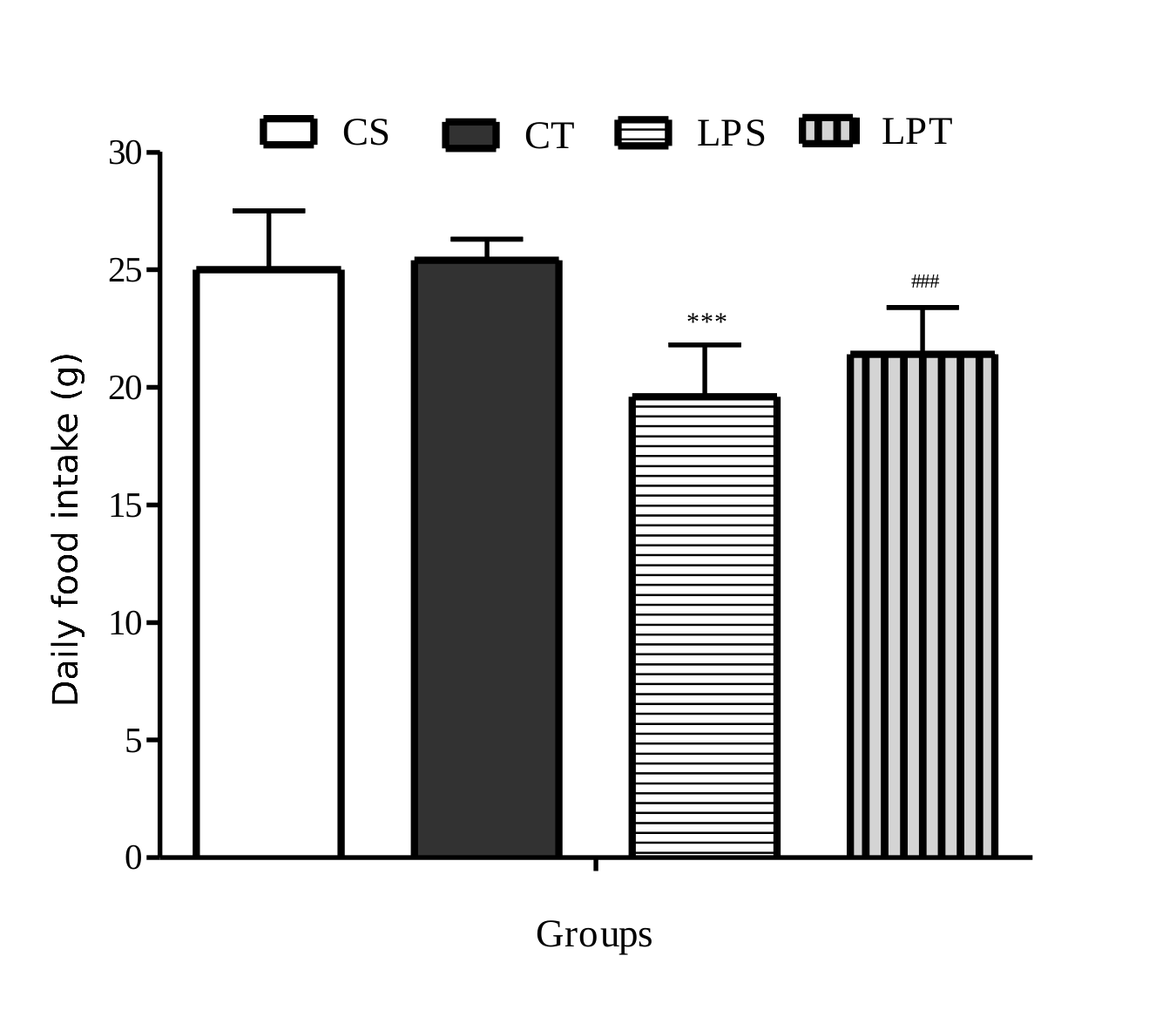

Supplement: Figure S1 — Daily food intake of pups between day-39 and day-42, from dams fed on low-protein or control diet during perinatal period. Rat pups received a daily bolus of L-tryptophan or Saline from day-12 to day-21. On rat pups from mothers fed on low protein diet, daily food intake was significantly different from control, irrespectively to L-tryptophan supplementation (Low-Protein Tryptophan (LPT, n = 9 pups); Low protein Saline (LPS, n = 9 pups); Control Tryptophan (CT, n = 12 pups), Control Saline (CS as white) *P<0.05 ***P<0.0001 by one way ANOVA followed by Bonferroni test. Data are expressed as means ±SEM (*LPS vs CS and #LPT vs CT.). (TIF) [file pone.0056231.s001.tif]

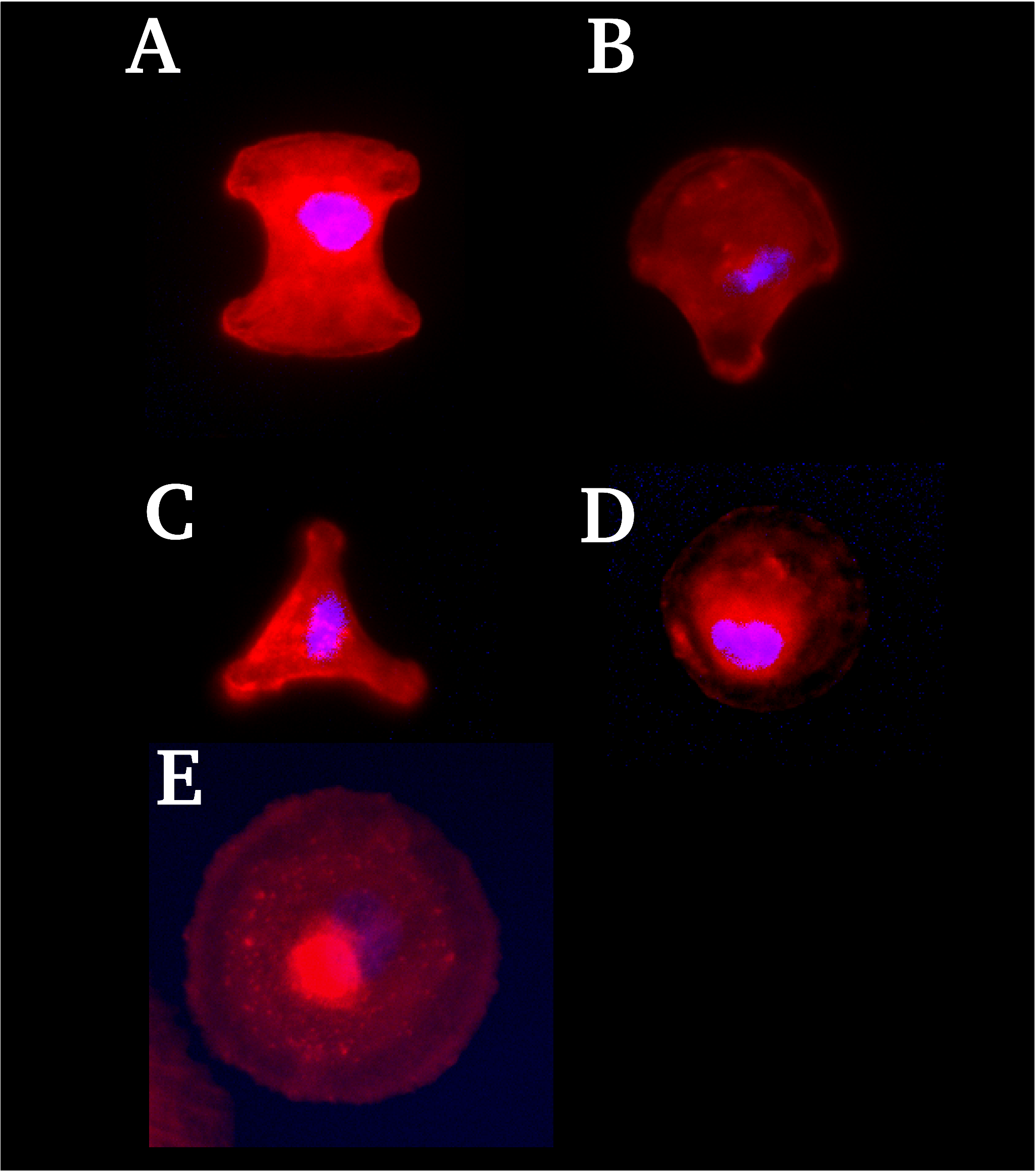

Supplement: Figure S2 — Morphology of representative primary cells, all expressing tryptophan hydroxylase (red) on Cytoo well preset with a wide array of forms. Similar diversity of phenotypes (A to E) was found on primary cells isolated from rat submitted to perinatal undernutrition or not. Supplementation with a bolus of L-tryptophan did not alter the phenotypes. (TIF) [file pone.0056231.s002.tif]

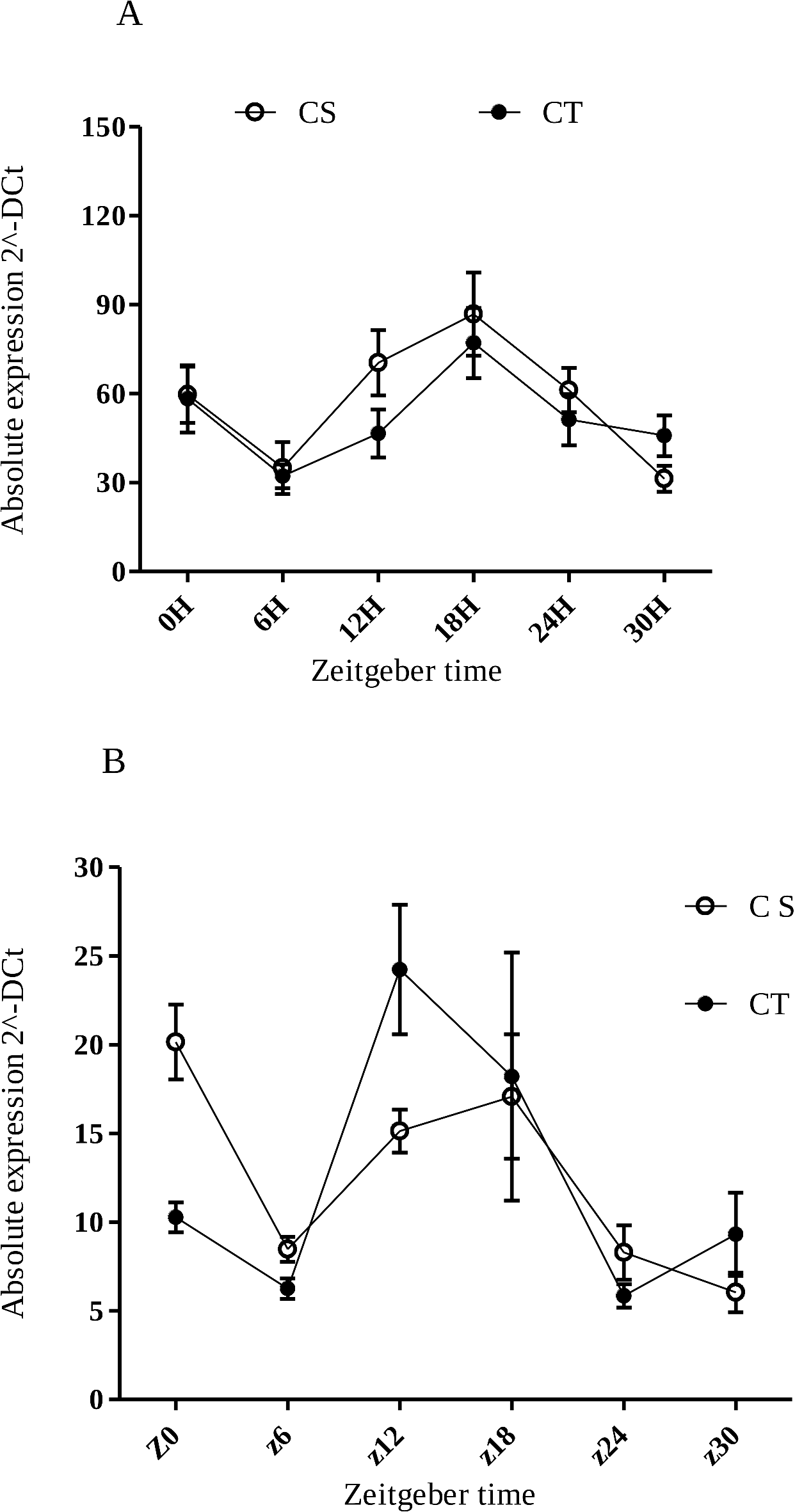

Supplement: Figure S4 — Evolution of mRNA expression of period1 (circle) and bmal1 (triangle) after a serum shock of cell lines obtained from at least 3 rats of the control group receiving a daily bolus of L-tryptophan (closed symbol) or a saline solution (open circle). Expression of circadian clock Per1 (A) and BMAL1 (B) transcripts in primary cultures from tryptophan (black square) and saline (white circle) rat offspring from mother fed on control diet. The transcript levels at 6 h intervals were measured by quantititative PCR and synchronized to time 0 h by fetal calf serum. Graphs represent the relative transcriptional level of genes averaged over at least 4 independent samples isolated by offspring of 45 d old derived from dams fed control diet and supplemented or not wit L-tryptophan early 12 d old at 21 d old. Each point corresponds to the means ±S.E.M. expression levels of 4–6 cells by groups (two-way ANOVA followed by Bonferroni test). (TIF) [file pone.0056231.s004.tif]

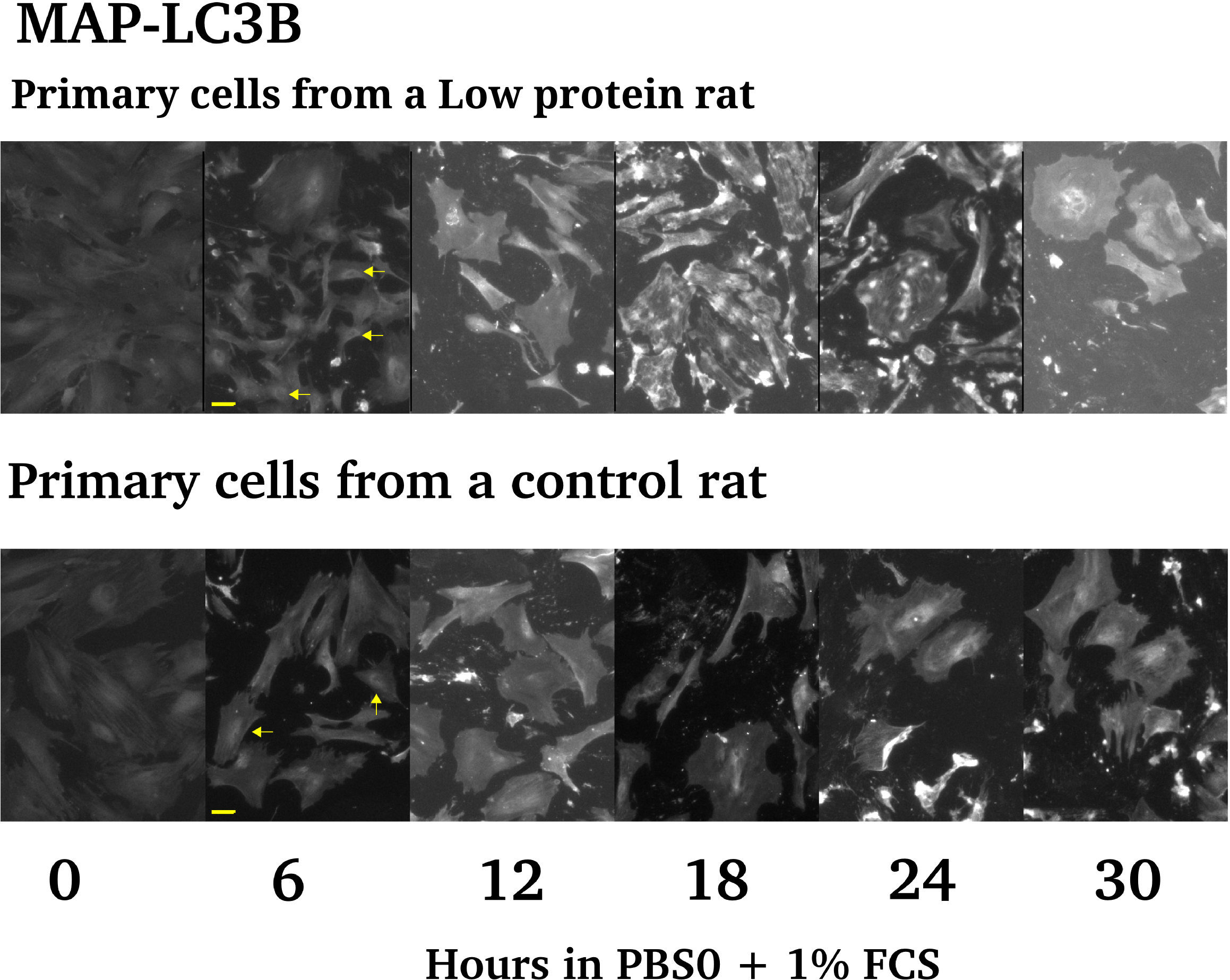

Supplement: Figure S5 — Expression of MAP LC3B protein in representative cultures of primary cells from rats with perinatal undernutrition or control-fed. Autophagosomes were clearly labeled after 6 under starving conditions (yellow arrows). Note that the density of cells are equivalent up to 12 h, thereafter the density of cells isolated from the rat with perinatal undernutrition is higher than the density of control. All cultures were made of surviving cells at 30 h and were not used for quantification. (TIF) [file pone.0056231.s005.tif]

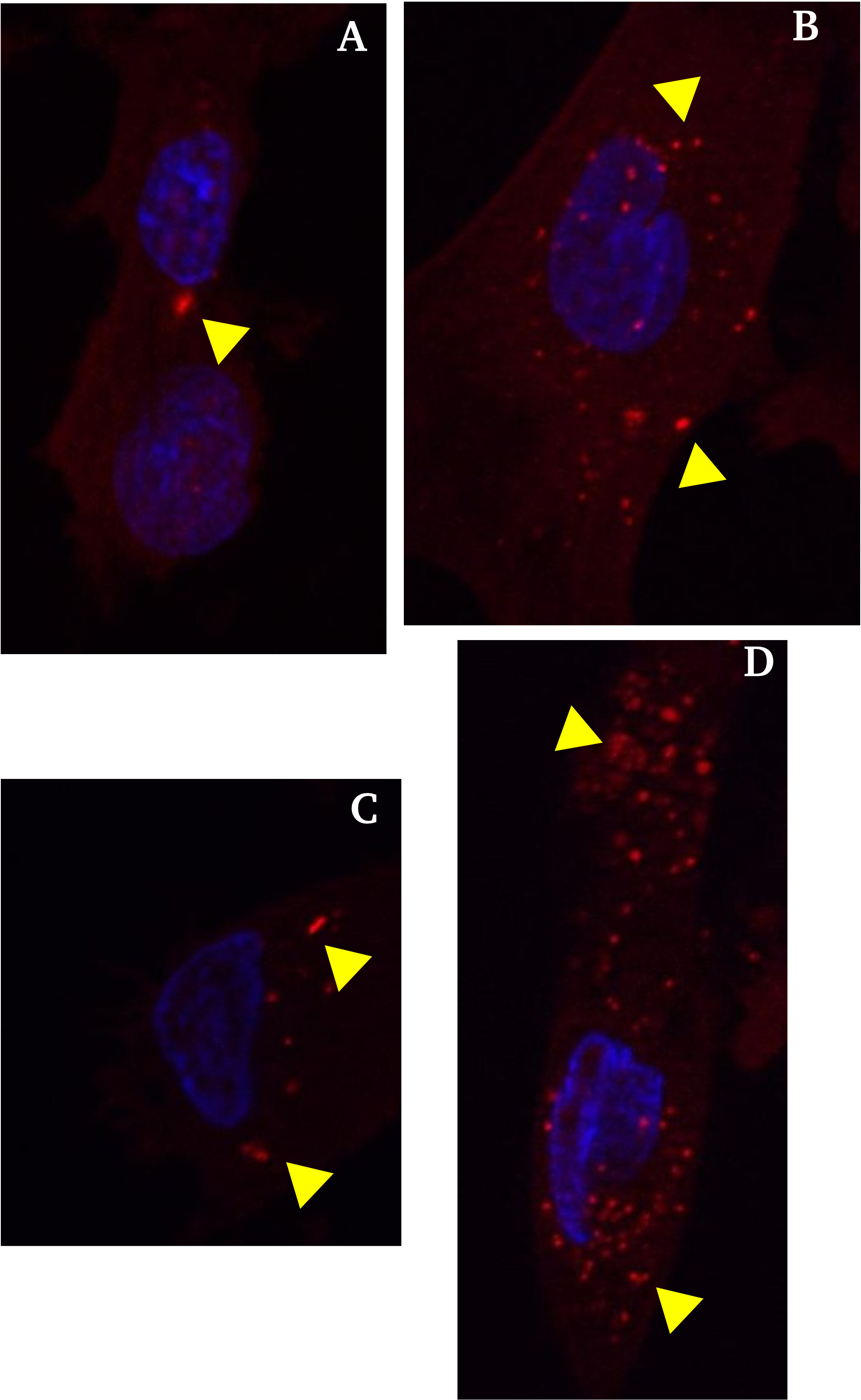

Supplement: Figure S6 — Expression of the chimeric LC3B-FP protein after infection of primary cells by a baculovirus construction and 4-hour starvation. Cells were isolated during the hyperphagic period from undernourished rats receiving daily bolus of L-tryptophan (A) or saline solution (B) and from control-fed rats receiving a daily bolus of L-tryptophan (C) or saline solution (D). Note that the number of autophagosomes labeled in red (yellow arrowheads) are equivalent between infected cells. (TIF) [file pone.0056231.s006.tif]
